# Pain Symptoms in Optic Neuritis

**DOI:** 10.3389/fpain.2022.865032

**Published:** 2022-04-14

**Authors:** Xiayin Yang, Xuefen Li, Mengying Lai, Jincui Wang, Shaoying Tan, Henry Ho-lung Chan

**Affiliations:** ^1^School of Optometry, The Hong Kong Polytechnic University, Kowloon, Hong Kong SAR, China; ^2^Department of Ophthalmology, The First Affiliated Hospital of the Medical College of Shantou University, Shantou, China; ^3^Department of Vascular Neurosurgery, The First Affiliated Hospital of the Medical College of Shantou University, Shantou, China; ^4^Shantou University Medical College, Guangdong, China; ^5^Department of Ophthalmology, The First Medical Center of Chinese PLA General Hospital, Beijing, China; ^6^Research Centre for SHARP Vision (RCSV), The Hong Kong Polytechnic University, Kowloon, Hong Kong SAR, China; ^7^Center for Eye and Vision Research (CEVR), Hong Kong, Hong Kong SAR, China; ^8^University Research Facilities in Behavioral and Systems Neuroscience (UBSN), The Hong Kong Polytechnic University, Hong Kong, Hong Kong SAR, China

**Keywords:** pain, optic neuritis, mechanism, treatment, multiple sclerosis, neuromyelitis optica spectrum disorder, myelin oligodendrocyte glycoprotein associated disease

## Abstract

Signs and symptoms of optic neuritis (ON), an autoimmune disorder of the central nervous system (CNS), differ between patients. Pain, which is commonly reported by ON patients, may be the major reason for some patients to visit the clinic. This article reviews the presence of pain related to ON with respect to underlying disorders, including multiple sclerosis (MS), neuromyelitis optica spectrum disorder (NMOSD), and myelin oligodendrocyte glycoprotein associated disease (MOGAD). The aim of this review is to provide an overview of pain symptoms in accordance with the context of various pathophysiological explanations, assist in differential diagnosis of ON patients, especially at the onset of disease, and make recommendations to aid physicians make decisions for follow up diagnostic examinations.

## Introduction

Optic neuritis (ON) is an optic neuropathy caused by inflammation. ON can arise from several etiologies, including multiple sclerosis (MS), neuromyelitis optica spectrum disorder (NMOSD), myelin oligodendrocyte glycoprotein antibody associated disease (MOGAD), acute disseminated encephalomyelitis (ADEM), and some systemic autoimmune diseases. Usually, ON presents as a symptom of an episode of one of several inflammatory diseases occurring in the central nervous system (CNS). It may, however, emerge as an isolated disease unity. Prompt and intensive therapy is required to control the development of disease, restore the comfort of the patient, and reduce relapses of disease based on accurate and rapid diagnosis of background diseases. Optic nerve atrophy may lead to a permanent visual loss in severe cases. This poor prognosis of ON in some cases highlights the importance of correct diagnosis of ON. Baseline data to consider for diagnosis include age, gender, visual loss, absence or presence of pain, and auxiliary tests. Consideration of follow-up tests should be guided by a detailed evaluation of symptoms, especially for patients that otherwise appear healthy.

### Objectives of Review

Phenotypes of ON associated with various etiologies overlap and, thus, careful assessment of their difference is required, since typical ON responds well to steroid therapy, whilst atypical ON is resistant to steroid therapy and tends to relapse. Although a published review has provided an introduction of prevalence and the underlying mechanism of pain in NMOSD and MOGAD without focusing on pain related to other etiologies, they were concerned primarily with systemic pain, especially neuropathic pain, which was highly relevant to the quality of life and paid less attention to ON related pain. The major purpose was to analyze how to improve the quality of life of patients (1). There have been limited reports concentrating on the symptoms of pain in ON and there is a lack of an extensive clinical database of prevalence of pain in different types of ON. This review focuses on the pain experienced by ON patients according to different disease etiologies, explores the mechanism of pain in ON, and summarizes the prevalence of different pain manifestations, including ocular pain, pain with eye movement, and headache. Treatments for pain for ON patients are also suggested.

### Method of Review

Relevant articles were identified by a search of of PubMed, by inputting the terms “optic neuritis” AND “pain”, “optic neuritis” AND “classification”, “multiple sclerosis” AND “pain”, “neuromyelitis optica” AND “pain”, “myelin oligodendrocyte glycoprotein AND “pain”, “pain with eye movement” and “treatment” AND “pain related to ON”. Only English language publications were included in the review. Titles and abstracts were reviewed to determine which articles were relevant. The full content of studies deemed relevant was subjected to detailed review. The references of each article reviewed were carefully examined to identify additional related publications within this field.

### Results of Review

A total of over 47 articles, including 21 original studies and 26 reviews were identified and were included in this article. The overview of the cited articles related to each sections below was summarized in Table 1.

**Table 1:** Summary of cited article in each session.

**Section**	**Type of cited articles**	**Content of the cited articles**	**References**	**Number of cited reviews and original studies**
Objective of the review	Review	The quality of life in patients with NMOSD	(1)	1 review
Different types of ON and the characteristics	Original	How to diagnose definite MS (99 subjects)	(2)	12 original studies and 5 reviews
	Review	Around 50% MS would be affected by ON	(3)	
	Review	The definition of clinically isolated syndrome	(4)	
	Review	White matter lesions increase risks of converting to MS	(5)	
	Original	Prognosis of visual acuity is better in MS (438 patients)	(6)	
	Original	Total 448 subjects were recruited. Better visual prognosis was discovered. Around one third of patients was found to be optic swelling	(7)	
	Original	Total 128 patients were recruited. Visual acuity became worse with aging	(8)	
	Original	Estimated 128 subjects were included. It took ~8 weeks to recover	(6)	
	Review	Bilaterality were more common in NMOSD	(9)	
	Guideline	Lesions in spinal cord are long and extensive in NMOSD	(10)	
	Review	Oligoclonal bands were found to be more frequent in MS	(11)	
	Original	AQP4-Ab is specific to NMOSD. This research included 148 subjects	(12)	
	Original	Glucocorticoids is necessary for treatment of NMO	(13)	
	Case report	Assessing CRION	(14)	
	Original	This research included 99 subjects	(15)	
	Original	Scotoma was found commonly in 448 ON subjects.	(16)	
	Original	Scotoma was the most frequent pattern of visual field defect. (99 subjects)	(2)	
Genetic factors of MS and NMOSD	Original	High risk of suffering ON for patients with familial history (25 subjects)	(17)	4 original studies and 1 review.
	Review	Higher risk to develop ON for patients with familial history	(18)	
	Original	HLA-DRB alleles were associated with higher rate of MS and NMOSD (17 NMOSD, 29 MS and 28 HC subjects)	(19)	
	Original	Non-MHC alleles were also found to associated with ON (110 NMOSD patients and 332 HC)	(20)	
	Original	Rs117026326 was associated with ON (144 NMOSD patients; 168 MS patients and 1403 HC)	(21)	
Pain symptoms associated with ON	Review	Pain can occur before or after damage of visual function	(5)	5 reviews and 8 original studies.
	Review	Two to three days before vision loss	(22)	
	Original	Pain with eye movement (128 subjects)	(6)	
	Original	Pain with eye movement was more common when the orbital segment of optic nerve was affected. (Total 95 subjects with acute ON were recruited)	(23)	
	Review	Lack of pain in ON when only intracranial part of optic nerve was affected	(24)	
	Original	Contraction of extraocular muscles (101 eyes with optic neuropathy)	(25)	
	Original	In one study with 48 subjects (21 seropositive for MOG-Ab, 27 seropositive for AQP4-Ab), MOG-Ab (+) patients are more likely to have anterior lesions of optic nerve	(26)	
	Original	AQP4-Ab are more likely to attack optic chiasm and tract (50 subjects)	(27)	
	Original	AQP4-Ab are more likely to attack optic chiasm and tract (163 subjects)	(28)	
	Original	Headache is more common in MOG-ON than NMOSD-ON (129 MOG-Ab positive patients)	(1)	
	Review	Headache related to ON	(22)	
	Original	MOG-Ab was more likely to involve optic nerve sheath	(29)	
	Review	Some chemokines related to headache in ON patients	(22)	
Mechanism of pain	Review	Neuropathic pain	(30)	11 reviews and 1 original study.
	Review	Glutamate is involved in pain experience	(31)	
	Review	Trigeminal nerve	(32)	
	Review	Trigeminal brainstem complex	(33)	
	Original	Nociceptive pain occurrence (50 subjects)	(29)	
	Review	Nociceptive pain	(34)	
	Review	Pain classification in MS	(35)	
	Review	The nervi nervorum	(36)	
	Review	T cells mediate the paralysis of AQP4 (+) patients	(37)	
	Review	Macrophage infiltration	(38)	
	Review	Mast cells in NMOSD	(39)	
	Review	Glutamate in pain	(40)	
The management of pain in ON patients	Original	Corticosteroids treatment for ON (750 participants in 6 randomly controlled trials)	(41)	Total 5 original studies and 2 reviews.
	Original	Plasma exchange (43 patients with 96 attacks)	(42)	
	Review	Immunomodulatory treatment	(43)	
	Original	Tocilizumab	(44)	
	Original	Spinal cord injury pain treatment by interferon-6	(45)	
	Original	Mofetil (90 subjects)	(46)	
	review	Cannabinoids for eye pain	(47)	
Comorbidity	Original	Thirty-five out of 67 subjects with MS and NMOSD suffered from depression.	(48)	3 original studies and 1 review.
	Original	Severe depression in NMOSD (71 subjects)	(49)	
	Review	GABA	(50)	
	Original	GABA transporter subtype	(51)	

*NMOSD, neuromyelitis spectrum disorder; MS, multiple sclerosis; AQP4-Ab, aquaporin 4 antibody; CRION, chronic relapsing inflammatory optic neuropathy; HC, healthy controls;*

*MOG-Ab, myelin oligodendrocyte glycoprotein antibodies; GABA, aminobutyric acid*.

## Different Types of ON and The Clinical Characteristics

ON is commonly recognized to be related to MS. Magnetic resonance imaging (MRI) plays a paramount role in diagnosis of MS. If an ON patient manifests a normal MRI, the conversion rate to MS is only 25%. In contrast, if hyperintense lesions are observed on the T2 sequence MRI, the conversion rate to MS is 75%. The typical lesions of MS are multifocal, sporadic, and demarcated (52). Over half of the ON patients involved in the Optic Neuritis Treatment Trial (ONTT) were affected by MS (52). Isolated ON and MS-associated ON (MS-ON) are usually grouped together as typical ON, since they share concurrent characteristics, including age ranging from 18 to 50, unilaterality, pain with eye movement, rapid vision loss, and the tendency to reach the lowest level of visual acuity within two weeks of onset. Clinically isolated syndrome (CIS) refers to that the patient underwent a single or multiple phases of neurologic symptoms that might progressed to MS but at that moment these symptoms cannot fulfill the diagnostic criteria of MS (Karussis, 2014). White matter lesions observed in MRI are mostly associated with MS (5). The recovery of visual acuity is always very good for MS patients, even without corticosteroid medication. Visual acuity of at least one-line was restored within three weeks for almost all patients without any treatment (53). Another study reported that 72% of impaired eyes achieved a complete recovery of visual acuity, while 92% recovered visual acuity better than 20/40 (7). The visual impairment of ON in older subjects (onset of ON later than 45 years old) is worse than that of their younger counterparts (8), often lasting for 8 weeks for a full recovery of the lesions of ON with frequent residual impairment (6). Atypical ON, especially the neuromyelitis optica spectrum disorder (NMOSD) associated ON (NMOSD-ON), is more commonly bilateral (around 20%) than MS-ON (9). In NMOSD, lesions in the spinal cord appear to be long and extensive, whilst the few lesions in the brain always lie in the hypothalamus and brain stem (10). For differentiated diagnosis, an oligoclonal band (OCB) is more frequently discovered in the cerebrospinal fluid (CSF) of MS patients (85%) than NMOSD patients (15-30%) (11). The presence of aquaporin protein 4 antibodies (AQP4-IgG), which are found in more than 90% of NMOSD-ON patients in conjunction with the clinical features, helps to diagnose NMOSD with high sensitivity (99%) and specificity (90%) (12, 54). Visual acuity in NMOSD-ON is relatively poor at the early stage of disease, with 80% of patients having visual acuity worse than 20/200. The recovery of vision is also reported to be very poor, with the visual acuity of ~30% of patients remaining at 20/200, with no further improvement (9). Of patients testing seronegative for AQP4-IgG, 20% tested positive for myelin oligodendrocyte glycoprotein (MOG) antibodies (MOG-IgG). Due to its unique clinical characteristics, including dominance of male gender, a single attack, better recovery of visual acuity, presentation as ON at onset is usually in adults, but presentation as acute demyelinating encephalomyelitis (ADEM) at onset is mostly in children. MOG associated disorder (MOGAD) is increasingly considered as a disease entity distinct from NMOSD. Unlike MS-ON, presentation with NMOSD-ON requires prompt therapy with glucocorticoids to relieve the damage to the optic nerve axons or immunomodulators to reduce the relapse rate (13). Recurrence of inflammation confined to the optic nerve in ON patients without fulfilling the criteria of MS-ON or NMOSD-ON is highly suggestive of a diagnosis of chronic relapsing inflammatory optic neuropathy (CRION). CRION responds well to both corticosteroid and immunomodulatory agents, but cessation of medication often results in relapse of disease (14). The patterns of visual field defect in these patients are variable and include scotomas, arcuate defects, and nasal step defects. Scotomas were the most common defects reported if eyes were examined by perimetry with a large field (2, 15, 16). Relative afferent pupillary defect (RAPD) is often present in patients with a monocular deficit. Optic swelling arises in only one third of patients (7).

Table 2 generalize several disease entities related to ON and summarize the clinical features of them.

**Table 2:** Clinical features of different disease entities that may develop ON.

	**Bilaterality**	**Severity and recurrence**	**Susceptible population**	**Visual acuity**	**MRI**	**Others**	**References**
MS-ON	Unilateral in most cases	Mild to moderate; monophasic in most cases	Young (averaged 32 years old) Caucasian women	Always recover within one month	Demyelinated lesions found in brain MRI; The lesions always disseminate in space and time.	Retrobulbar ON (65%) remains the major ON. Good response to corticosteroid therapy.	(7, 53, 55–59)
NMOSD-ON	Bilateral in most cases	Severe; relapsing frequently	Older than MS patients (more patients >50 or <18 years old); Asian or African women	Progress to even no light perception	LETM: more than 3 segments; The lesion of ON always locates posteriorly and mostly involves optic tract and chiasm; Always involve more than 50% optic nerve.	Resistant to corticosteroid and immunosuppressant therapy. Accompanied by intractable nausea. Cell-based assay is most sensitive for AQP4-Ab detection (99%).	(7, 53, 55, 57, 59–63)
MOGAD-ON	Bilaterality accounts for 40%	Recurrent cases accounts for 80%-93%	Two study reported 31 years old. One study reported 40 years old; Caucasian women.	Good recovery of visual acuity compared to NMOSD-ON	Involvement of optic nerve in MRI always locate at the anterior segment.	Optic disc swelling is more pronounced than MS-ON. ON is often isolated.	(64–67)
CRION	Unilateral or bilateral	Relapsing course	Webb's study reported 71% female. The predilection for age and race is not obvious.	Good vision recovery and pain resolution after steroid treatment	Variable	Seronegative AQP4 and cannot satisfy the Macdonald's criteria.	(14, 68, 69)
ADEM-ON	Higher rate of bilaterality	Rare relapsing cases; always monophasic	Children less than 10 years old	Poor visual acuity	The margin of lesion is poorly defined; Greater lesions (1–2 cm) compared with other etiologies in white matter; most lesions are supposed to emerge at the same age compared to dissemination in space and time for MS.	Virus infection was considered to be the potential origin of this disease since it might initiate the procession of demyelination.	(70–73)

*MS, multiple sclerosis; NMOSD, neuromyelitis optica spectrum disorder; MOGAD, myelin oligodendrocyte glycoprotein; CRION, Chronic relapsing inflammatory optic neuritis; LETM, longitudinal extensive transverse myelitis; ON, optic neuritis; CIS, clinically isolated syndrome; AQP4-Ab, aquaporin-4 antibodies; ADEM, acute disseminated encephalomyelitis*.

## Genetic Factors of MS and NMOSD

MS and NMOSD are both sporadic diseases and the familiar segregations of them are compatible with non-Mendelian inheritance. It suggested that rare relatives of MS or NMOSD were affected by the same condition. However, the susceptibility of individuals with MS or NMOSD family members is higher than that of the general population. (17, 18) Previous screening of human leukocyte antigens (HLA) class II DR genes found that HLA-DRB1^*^15 (Odds ratio value = 15.89) and DRB5 alleles were more likely to be represented in MS patients. On the other hand, HLA-DRB1^*^03 (Odds ratios value = 3.23) and DRB3 alleles were associated with the emergence of NMOSD (19). In another study investigating non-major histocompatibility complex (non-MHC) genes, it screened the susceptibility of 35 MS-related loci among 110 NMOSD patients, but no significant higher risk of developing NMOSD in patients with these predisposing loci can be found compared with 332 normal subjects (20).

One genotyping research conducted its investigation on HLA-DRB1 and DPB1 alleles in 211 Japanese NMOSD patients compared with 1,919 normal healthy subjects as controls. A single nucleotide polymorphism (SNP) rs1964995 located in MHC region was reported to be related to occurrence of NMOSD (OR value = 2.33). Two risk alleles (HLA-DRB1^*^08:02 and HLA-DRB1^*^16:02) were also detected by the same research while HLA-DRB1^*^9:01 was found to be a protective factor. One more to mention in this study was that a SNP rs1516512 in gene potassium calcium-activated channel subfamily M alpha 1 (KCNMA1) correlated with disability and transverse myelitis. Immunohistochemistry also found KCNMA1 with the area of endfeet of astrocytes where most NMOSD lesions located (74). In a Chinese Han cohort, rs117026326 was screened in 144 NMOSD patients, 168 MS patients and 1,403 healthy controls due to that NMOSD always coexists with some systemic autoimmune diseases Sjogren's syndrome, systemic lupus erythematous (SLE) and rheumatoid arthritis (RA) associated with overrepresentation of rs117026326. The result revealed that rs117026326 in the upstream of general transcription factor II-I (GTF-2I) gene could also be the risk factor of developing NMOSD (OR value = 2.535) (21). However, no significant association between MS and rs117026326 was detected compared with controls. Overall, from mentioned above, MS and NMOSD are supposed to be two entities of disease with distinct genetic background.

## Pain Symptoms Associated With ON

Due to complex associated ON diseases, pain presents with multiple characteristics. Pain can be the symptom indicating ON especially when this pain experience is following eye movements. Besides pain related to ON, NMOSD-ON and MS-ON patients also share similar systemic pain symptoms according to NMOSD and MS. Table 3 shows and summarizes the pain arising from non-ocular courses. Typical ON patients are more likely to experience mild periocular pain and pain following eye movement. Experience of such pain is relieved only after several days. Pain can either precede or occur after the visual loss (5), although pain usually occurs 2–3 days before the impairment of visual function (22). The duration of pain with eye movement usually persists for less than one week (6). The ONTT reported a high rate of ocular and periocular pain (92%) and pain following eye movement (87%) (7). Pain was worsened by eye movement (90%), while the orbital segment is enhanced on MRI after gadolinium injection (23). Lack of pain following eye movement is always related to the lesion being located in the intracranial part of the optic nerve (79). Fazzone et al. (23) also reported that pain with eye movement is presumably absent if the lesion is confined to the intracranial and canalicular part of the optic nerve. Pain arises from different mechanisms when different portions of the optic nerve are involved.

**Table 3:** Systemic pain symptoms in MS and NMOSD.

	**Systemic pain**	**Life quality**	**References**
MS	Seventy-five percentage of patients suffered from pain due to a range of courses. For treatment of MS, 30% medical treatments were be contributed to pain relief.	Fatigue was frequent and some patients were considered to have mental disorder. Family lives were also influenced by disease.	(75)
NMOSD	Mechanical allodynia and thermal hyperalgesia both make up of neuropathic pain which occurred frequently in NMOSD. Pain was not sufficiently controlled in NMOSD since NMOSD lesions were more likely to affect spinal cord where the nociceptor signaling pathway went through.	Depressive status and fatigue became more common than MS. The scores of Beck depression inventory were higher than that in MS.	(76–78)

### Ocular Pain

Sometimes, patients may mistakenly indicate the site of their lesions because ocular pain can radiate to the periocular area, since the whole orbital structure shares the same innervation of the first division of the trigeminal nerve. It may be postulated that the pain sensation radiates from the eye to the periocular area or other regions of the orbital structure (24). It is very common for ON patients to complain that they suffer from ocular, periocular, and retroocular pain. Table 4 summarize the incidence of retrobulbar pain and pain following eye movement in several studies.

**Table 4:** Overview of researches with respect to the incidence of ocular pain and pain with eye movement in ON patients.

**The population**	**Incidence of ocular pain or pain with eye movement**	**Other implication in the article**	**Reference**
The population in Central Europe	Estimated 92% of ON patients suffered from pain with eye movement in a cohort of 468 CIS subjects	The incidence of ON in general population was 5/100,000. Seventy percent of ON patients were female. The percentage of bilaterality was only 0.4%. Mean age was 39 years old. Female account for 68.8%. The pattern of incidence in both CIS and MS were alike according to sex and race.	(80)
Japanese cohort	The incidence of pain with eye movement was reported to be 77% in MOG-Ab positive cohort.	Seronegative AQP4-Ab and MOG-Ab was found in 77% of 531 patients. The proportion of pain with eye movement was close to that of optic disc swelling.	(81)
The Unites States cohort	ONTT in 1991 reported a rate of 92% for pain with eye movement with regard to 448 eligible subjects. The rate of pain following eye movement was reported to be 87%	Pain with eye movement was not related to optic disc swelling; Up to 77.2% patients were female. The mean age was 31.8 years old. Optic disc swelling was found in 35.3% of total subjects. Demyelinated lesions were found in 48.7% patients.	(7)
The Unites States	In 96 subjects, no enhancement on optic nerve in MRI was found in 5 patients. As for ON patients with enhancement on optic nerve in MRI, 67 patients out of 91 (73.63%) suffered from pain with eye movement.	Pain with eye movement occurred more frequently in patients with enhancement of orbital segment in MRI than without enhancement of orbital segment in MRI.	(23)
Korean cohort	Eighty-nine percent (8 out of 9) eyes affected by ON was related to pain with eye movement	Six out of 9 eyes (66%) were affected by optic disc swelling. On the last follow-up session, the averaged visual acuity of all patients could recover to 20/20.	(82)

*CIS, clinically isolated syndrome; ON, optic neuritis; MOG-Ab, myelin oligodendrocytes glycoprotein- antibody; AQP4 4-Ab, aquaporin 4- antibody; ONTT, optic neuritis treatment trial; MRI, magnetic resonance imaging*.

### Pain With Eye Movement

**Figure 1:**
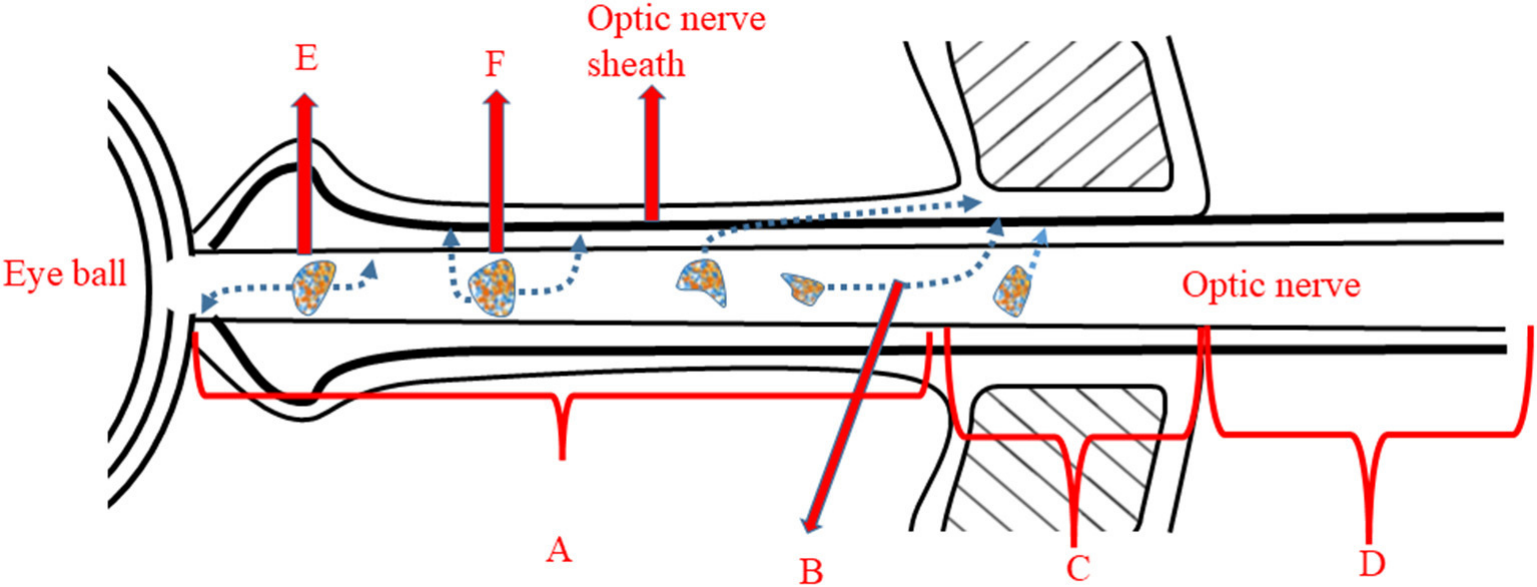
Illustration of origin of pain with eye movement and headache. A, Orbital segment of optic nerve: lesions lying in this segment often trigger pain with eye movement due to proximity to site B; B, The optic nerve sheath attaches to the ocular muscles at this site. Movement of the eye induces the pain experience. C, Intracanalicular portion of the optic nerve. D, Intracranial segment of the optic nerve. E, A mild lesion within the orbital segment which is unlikely to infiltrate to the optic nerve sheath. F, A severe lesion within the orbital segment, which will penetrate the optic nerve sheath and lead to headache.

Pain sensation worsening after movement of the eye ball is specific to typical ON. At the site of the apex, the optic nerve sheath is proximal to the superior and medial rectus. Consequently, when the patient moves the eye ball, contraction of the ocular muscles irritates the inflamed optic nerve sheath to cause the pain (25) (Figure 1). It is less likely for patients with anterior ON to develop pain with eye movement, because the optic nerve sheath of the anterior nerve is not pulled by the ocular muscles. In a study involving 91 patients, the frequency of ocular pain and pain with eye movement was higher for patients with enhanced orbital segment in MRI, whilst patients without an enhanced orbital segment were more likely not to experience any painful sensation (23) (Table 4).

A recent study showed that MOGAD related ON (MOGAD-ON) predominantly affects the anterior segment of the optic nerve, especially the optic nerve head (ONH), whereas the posterior segment of the optic nerve is most commonly involved in patients with seropositive AQP4-IgG (26). Consistent results were reported by other studies, which observed that ON with positive AQP4-IgG mostly involved the optic chiasm and optic tract (27, 28). This may explain why the frequency of eye pain is relatively lower in NMOSD-ON patients (67%) than in MOGAD-ON (1). A prospective study could analyze whether patients with MOGAD-ON and ON with positive AQP4-IgG manifest a lower proportion of pain with eye movement because the original lesions of both ON reside distally to the mobile portion of the orbital apex (Figure 1).

### Headache

Apart from ocular pain and pain following eye movement, headache is another common symptom related to ON, especially if the optic nerve sheath is severely involved (22). Headache can be related to both an ocular or a non-ocular cause, the latter varying in both MS and NMOSD patients. The pain may arise from a cervicogenic source, while the lesions remain at the cervical segment of the spinal cord. Alternatively, sensitization of the trigeminal nerve system may produce spontaneous pain in some cases. Therefore, it is evident that MRI scanning to locate the lesions in MS or NMOSD is necessary to differentiate distinct etiologies of headache. The current section only concerns headache related to ON.

The lesions of MOGAD-ON are more likely to be located at the orbital segment of the optic nerve, inducing the swelling of the optic nerve sheath seen using MRI (27–29). However, the relationship between the extent of oedema in the optic nerve sheath and severity of pain remains controversial. A recent retrospective study of headache related to ON, reported that 50.5% of MOGAD-ON patients had symptoms of headache as opposed to only 14% of MS patients presenting with a headache (1). Visual impairment following headache is profound for MOGAD-ON patients. Fifteen of the 64 patients who underwent MRI investigation were found to have lesions involving the anterior segment of the optic nerve, of whom nine had lesions extending to the intracanalicular segment. The researchers concluded that headache and ensuing visual impairment could be prodromal symptoms for MOGAD-ON hence, stressing the necessity of an MOG-IgG test. They also proposed that inflammation involving the anterior segment of the optic nerve in MOGAD-ON is likely to be the cause of the high rate of headache and visual loss observed in this condition: the more severe the inflammatory attack on the optic nerve sheath surrounding the optic nerve, the higher the possibility that the patient will suffer from headache (1) (Figure 1). This retrospective study, however, may have underestimated the proportion of headache with MOGAD-ON, since some physicians did not ask whether the patient had a headache. A longitudinal study is essential to investigate the relation of types of headaches, such as migraine-like headache, pulsive headache, or mild headache, to locations of affected segments of the optic nerve. Table 5 listed all headaches symptoms due to brain or spinal cord lesions for physician to differentiate pain related to ON from pain due to various etiologies.

**Table 5:** Overview of headache in patients with MS or NMOSD.

	**Overview of headache in MS or NMOSD**	**Type of headache**	**Incidence of different types of headaches**	**Potential affected area according to different types of headaches**	**Others**	**References**
MS	Up to 38.9% during the process of relapse compared to only 3.5% during remission	Headache related to optic neuritis	Up to 14% MS patients reported ON associated headache	Optic nerve	/	(1)
		Tension headache	Commonest	/	/	(83–86)
		Migraine	High incidence in MS	Substantia nigra and red nucleus	May indicate high rate of relapsing-remitting course	
NMOSD	More severe than MS reported by Kanamori	Paroxysmal painful tonic spasms	Account for 90% NMO patients	Myelitis	/	(87–89)
		Ocular pain	Retro-orbital pain was present in 50% of patients	Optic neuritis	Simultaneous onset of bilateral sides was more frequent.	(89)
		Trigeminal neuralgia	In a cohort of 258 NMO patients, 3 patients (2.5%) were reported to suffer from trigeminal neuralgia	The second and third division of trigeminal nerve	Electric like, severe and short-lived pain	(11, 90)
		Occipital headache (radiate to the back of neck)	/	Cervical segment of spinal cord	Correspond to the musculoskeletal dysfunction	(91, 92)
		TAC	/	Trigeminal nerve and upper segments of spinal cord	Estimated 8 times for one day	(93, 94)
		Headache with fever	/	Encephalitis	The onset of headache is accompanied by low-degrade fever, nausea and intractable vomiting	(95)

*MS, multiple sclerosis; ON, optic neuritis; NMO, neuromyelitis optica; NMOSD, neuromyelitis optica spectrum disorder; LETM, longitudinal extensive transverse myelitis; TAC, trigeminal autonomic cephalalgia*.

Pentraxin-3 and interleukin-6 (IL-6) have already been recognized as biomarkers for headache syndromes (22). Pentraxin-3, measured in serum, is related to immune reaction in autoimmune diseases and has been reported to increase to a high level in patients with headache. IL-6 in CSF has been shown to be another pro-inflammatory factor able to predict the inflammatory status in NMOSD. IL-6 is also suggested to be linked with headache, especially migraine attacks (22).

## Mechanism of the Pain Associated With ON

The sensory pain components, nociceptive pain and neuropathic pain, are generated via different pathways. For the nociceptive pain, action potential is produced upon activation of nociceptors and transmitted along the sensory pathway to the CNS. Influxes of sodium and calcium cause depolarization of action potential to initiate neural firing. The opening of sodium and calcium gates is mediated by transducer proteins, including G-protein-coupled channels, ion linked channels, and tyrosine kinase linked channels. To partially regulate the transmission of action potential, hyperpolarization of the nerve is caused by potassium channels that respond to overload of voltage, calcium, and adenosine triphosphate (ATP). In contrast, neuropathic pain is caused by sensitization of transmission. This condition is attributed to dysfunction or turbulence of the nervous system. Spontaneous pain and hyperalgesia reflect features of neuropathic pain (30).

With regards to pain associated with ON, the optic nerve itself is not able to convey pain information. Thus, the origin of this pain is always from the meninges covering the optic nerve. Sporadic nociceptors within the meninges respond to noxious stimuli and deliver impulses to surrounding axons.

Initially, nervi nervorum, a group of unmyelinated fibers, innervate the territories of the optic nerve sheath and receive the nociceptive stimuli from nociceptors in the peripheral tissues. Peripheral nervi nervorum also mediate chronic pain if the axons are involved (36). Polymodal nociceptors can respond to different stimuli due to diverse expression of ion channels of the trigeminal ganglion (TG). The ion channels of Piezo 2, the transient receptor potential vanilloid 1 (TRPV1), the transient receptor potential ankyrin 1 (TRPA1), and the transient receptor potential melastatin 8 (TRPM8) correspond to sensations of mechanical forces, heat, chemical agents, and coldness, respectively. The sensitivity and excitability increase, while the terminals of axons within the TG are involved by lesions. Subsequently, these ion channels are modulated to reduce the pain threshold of nerve membranes, which cause persistent pain (96). Central sensitization processed in nociceptors also facilitates hyperalgesia of the eye due to the continuous input of pain, leading to the frequent combination of glutamate and its receptors (31). The trigeminal nerve can be sensitized and activated to cause neuropathic pain and pain enhancement, by which it becomes susceptible to noxious stimuli (32). Subsequently, the axons of the TG are stimulated by noxious stimuli to transmit the pain information.

Finally, the TG sends the stimulus to the axons to end at the bodies of the trigeminal brainstem complex (33). Thus, the pain associated with ON arising from nociceptors in the meninges of the optic nerve constitute nociceptive pain (29). During the process of ON, mediators, including prostaglandin E2 (PGE2), tumor necrosis factor α (TNF-α), and interleukin-6 (IL-6), are released from the lesions of ON stimulated nociceptors within the orbital region (34).

However, it is unlikely that mediators of inflammation in ON infiltrate deeply and directly to stimulate the nervi nervorum. The less likely possibility of involvement of the nervous system, which produce neuropathic pain, has been described in an earlier review (35). Therefore, the trigeminal ganglion would be spared and central sensitization would be absent in ON. This may also explain why ON patients commonly complain of transient pain.

It has previously been reported that neuropathic pain rarely occurred in ON patients (1, 24, 27, 28). However, most of the ON patients in these studies experienced pain associated with MS, only a few having ambiguous etiologies. In contrast, NMOSD-ON pain seems to be more severe (36). NMOSD is mediated by the lymphocytes T helper (Th1 and Th17) (37). They may induce deaths of neurons, oligodendrocytes and astrocytes. Microglia cells phagocytose those cell debris and release chemokines, cytokines and reactive oxygen species (ROS) which is followed by recruitment of adhesion molecules. The speed of bloodstream is slowed down and cause high concentration of chemokines to facilitate production of macrophages. By presenting their components to T lymphocytes, macrophages initial the inflammation within the tissue (38). Following the initiation of inflammation in peripheral organs, mast cells are considered to be able to cross over the blood brain barrier (BBB) and release chemokines and tumor necrosis factor (TNF). Mast cells also generate proteinases Matrix metalloproteinases (MMP) 2 and MMP 9 to increase the permeability of the BBB and recruit more inflammatory cells into CNS system. TNF has already been demonstrated to be a strong pro-inflammatory factor that is capable of sensitization of nociceptors in meninges (39) Moreover, it is well known that NMOSD preferentially affects the astrocytes around the trigeminal nerve. Astrocyte re-uptake glutamate from the synaptic pool and convert it to glutamine by the excitatory amino acid transporters-2 (EAAT-2). In order to lower the concentration of glutamate within the cleft of synaptic contact, EAAT-2 was activated when suffering from an overdose of glutamate. Disorders of astrocytes will thus lead to a build-up of glutamate due to loss of EAAT-2. This excessive glutamate depolarizes the postsynaptic contact and, in turn, intensifies the pain experience (40). It remains uncertain whether NMOSD-ON or MOGAD-ON patients experience neuropathic pain. Some neuropathic pain may be neglected by physicians. Further studies are needed to investigate the long-erm pain symptoms of NMOSD-ON.

## The Management of Pain in ON

No studies have explored the function of potent analgesia and recommended their use for relief of pain, since this pain arises from the pathogenesis of ON and it is tolerable for the majority of patients. As mentioned previously, steroids are the first line therapy for ON, although the necessity for pain relief in typical ON still remains uncertain (41). Up to 500–1,000 mg intravenous administration of methylprednisolone (IVMP) is prescribed to inhibit inflammation of optic nerve. If this is not successful or the visual acuity does not recover after 3 days of IVMP, an additional 2 days are recommended to enhance the effect. For resistant cases, a double dose of IVMP is suggested. An alternative strategy for resistant cases is use of plasma exchange (PLEX) which aims to filter out autoantibodies throughout the circulation system. Five continuous cycles have been recommended to treat relapsing or resistant cases. Each cycle involves exchanging of one volume of plasma by 5% albumin (42).

Immunomodulatory agents have been shown to reduce the incidence of relapses (43). As described previously, IL-6 presumably gives rise to headaches in NMOSD and disrupts the CNS system, since it facilitates the activation of TH17 cells. Tocilizumab has been noted to be an antibody against the IL-6 receptor and its administration ameliorates the progression and pathological changes of NMOSD (44). It also relieves pain by turning down the glutamate transporter 1 (GLT1), which is associated with the depolarization of the post-synaptic contact (45).

Mycophenolate mofetil (MMF), which is suggested to be useful in the treatment of NMOSD and MOGAD, has been shown to inhibit the proliferation of B cells and T cells (46). It is acknowledged that patients with MOGAD rely strongly on a combination of steroid and MMF to relieve their pain (46).

Rituximab depletes B-cells by anchoring at the CD 20 epitope located on the surface of B cells and is effective in controlling the CNS damage in NMOSD (97). However, it is estimated that one third of MOGAD patients are refractory to rituximab. This population of patients relapse even after the administration of rituximab (98).

The endocannabinoid system is effective in modulating ocular pain and suppressing the inflammation with safer administration. It aims to bind to the cannabinoids-2 receptor (CB2R) and have an anti-inflammatory action. Current treatment with endocannabinoids is mainly confined to injuries of the cornea, such as alkali burns and dry eye. There is a lack of a detailed investigation into the effect on the pain related to optic neuritis. In addition, it has been noted that the pain modulation of endocannabinoids may be associated with inhibition of sensitization of TG. However, further research is needed to determine whether the biomarker L-type voltage gated Ca2+ channels are significantly inhibited in TG by endocannabinoids. Topical formulations of endocannabinoid are preferred to reduce systemic side effects (47).

## Comorbidity Due to MS and NMOSD

MS and NMOSD are always comorbid with emotional problems or disabilities that may cause some influence on their quality of life. In one previous study by Barzegar in 2021, 35 (52.2%) patients were affected by 44 comorbidities including 29 somatic, 13 psychiatric and 2 autoimmune disorders. Anxiety, migraine and depression disorder were all listed as commonest symptoms. With respect to those patients with AQP4-Ab (+), the frequency of suffering from psychiatric disorders is significant higher (48). Furthermore, the depression NMOSD patients suffered from was correlated with pain experience. Pain and depression coexisted to impose the influence on the quality of life (49). Activation of glutamatergic system and inactivation of GABAergic system were both considered to be mechanisms of developing depression in NMOSD (50, 51).

## Summary

Pain is a frequently reported symptom associated with ON. Pain symptoms differ between the various associated diseases and can be a useful diagnostic tool for identifying the type of disease. Bilateral severe orbital pain and headache with mild vision loss may indicate the diagnosis of MOGAD-ON. Pain followed by severe and irreversible visual loss as well as bilaterality suggests NMOSD-ON, while classical pain with eye movement, unilaterality, and reversible visual impairment can be an indicator of MS-ON. Pain of ON is initially triggered by activation of nociceptors in peripheral tissues. Through the progression of TG and the thalamus, the action potential caused by pain is projected to the cortex of the brain. Although pain can be aggravated by sensitization of the central nervous system, it remains uncertain whether the overreaction of the nervous system is evoked in ON patients. The transient and tolerable pain induced by ON does not require any analgesia to relieve. Pain can be resolved over a short period of time and by steroid treatment.

## Author Contributions

ST and HC: concept and design. XY, XL, ML, JW, ST, and HC: acquisition, analysis, and interpretation of data. XY: drafting of the manuscript and statistical analysis. ST and HC: revision of the manuscript. All authors had full access to all the data in the study and were responsible for the integrity and accuracy of data.

## Funding

This work was supported by the National Natural Science Foundation of China (Project Code: 81800822 to ST) and the Internal Research Grant from the Hong Kong Polytechnic University 2020-23 (Project Code: ZVS7 to ST). The funding organization had no role in the design or conduct of this research.

## Conflict of Interest

ST and HC are employed by Centre for Eye and Vision Research. The remaining authors declare that the research was conducted in the absence of any commercial or financial relationships that could be construed as a potential conflict of interest.

## Publisher's Note

All claims expressed in this article are solely those of the authors and do not necessarily represent those of their affiliated organizations, or those of the publisher, the editors and the reviewers. Any product that may be evaluated in this article, or claim that may be made by its manufacturer, is not guaranteed or endorsed by the publisher.
